# The Homomorphisms and Operations of Rough Groups

**DOI:** 10.1155/2014/507972

**Published:** 2014-06-05

**Authors:** Fei Li, Zhenliang Zhang

**Affiliations:** ^1^Department of Mathematics, Beijing Forestry University, Beijing 100083, China; ^2^Department of Mathematics, Kunming Institute of Technology, Kunming, Yunnan 650000, China

## Abstract

Based on the light relation between a normal subgroup and a complete congruence relation of a group, we consider the homomorphism problem of rough groups and rough quotient groups and investigate their operational properties. Some new results are obtained.

## 1. Introduction

Rough set theory, proposed by Pawlak [1], is an extension of set theory for study of information systems characterized by inexact and uncertain information. It has been demonstrated to be useful in fields such as knowledge discovery, data mining, decision analysis, pattern recognition, and algebra.

Rough set theory includes three basic elements: the universe set, the binary relations, and a subset described by a pair of ordinary sets. In the past few years, most studies have been focusing on the binary relations and the subsets; many interesting and constructive extensions to binary relations and the subsets have been proposed [1–8]. But the researchers have paid little attention to another basic element: the universe set. In real world, some universe has been given operations, such as the set of natural numbers and the set of real numbers. So, it is very natural to ask what would happen if we substitute an algebraic structure for the universe set. Biswas and Nanda [9] generalized the universe of rough sets to groups and introduced the notion of rough subgroups and some new properties of rough approximations have been obtained. Jiang et al. [10] investigated the product structure of fuzzy rough sets on a group and provided some new algebraic structures. Yin et al. [11] studied fuzzy roughness of *n*-ary hypergroups based on a complete residuated lattice. Xiao and Zhang [12] studied the rough sets on a semigroup and proposed two new algebraic structures—rough prime ideals and rough fuzzy prime ideals. In [6], a new algebraic definition for pseudo-Cayley graphs containing Cayley graphs has been proposed, a rough approximation was expanded to pseudo-Cayley graphs, and some new properties have been obtained. For more other papers on this line please refer to [12–24], which have greatly enriched the theoretical research of rough sets.

The aim of this paper is to investigate the homomorphism problem of rough group and rough quotient groups. The rest of the paper is organized as follows. In Section 2, we recall some basic notions and results which will be used throughout the paper. In Section 3, the homomorphism problems of rough groups and rough quotient groups are studied and some related properties are discussed. In Section 4, congruence relation and the operation of rough groups are investigated.

## 2. Preliminaries

Let *G* [25] be a group with unit element *e* and let *ρ* be an equivalence relation on *G*. If ∀(*ab*), (*cd*) ∈ *ρ* we have (*ac*, *bd*) ∈ *ρ*, then *ρ* is called a congruence relation and [*a*]_*ρ*_ indicates congruence class of *a* about *ρ*. Furthermore, if [*a*]_*ρ*_[*b*]_*ρ*_ = [*ab*]_*ρ*_, then *ρ* is called complete.

**Lemma 1 (see [1])**

Let *ρ* be a congruence relation of *G*. Then, *N*_*ρ*_ = {*a* ∈ *G* | (*a*, *e*) ∈ *ρ*} is a normal subgroup of *G* and (*a*, *b*) ∈ *ρ*⇔*a* ∈ *bN*_*ρ*_; contrarily, if *N* is a normal subgroup of *G*, one can define a congruence relation *ρ*_*N*_, where (*a*, *b*) ∈ *ρ*_*N*_⇔*a* ∈ *bN*_*ρ*_; then *ρ*_*N*_ is a congruence relation of *G*, and *N*_*ρ*_ = {*a* ∈ *G* | (*a*, *e*) ∈ *ρ*_*N*_}.

Lemma 1 indicates the congruence relation of *G* and the normal subgroup of *G* is one to one correspondence.

**Theorem 2**

Let *N* be a normal subgroup of *G*; then *ρ*_*N*_ is a complete congruence relation.

**Proof**

Consider ∀*a*, *b* ∈ *G*[*a*]_*ρ*_*N*__[*b*]_*ρ*_*N*__ = (*aN*)(*bN*) = *ab* *N* = [*ab*]_*ρ*_*N*__. Therefore, *ρ*_*N*_ is a complete congruence relation.

According to Theorem 2, we know the complete congruence relation of *G* and the normal subgroup of *G* is also one to one correspondence.

**Definition 3 (see [1])**

Let *ρ* be an equivalence relation on *G* and *A* a nonempty subset of *G*. Then, the sets

(1) $$\begin{array}{c} \underline{\rho }\left(A\right)=\cup \left\{{\left[x\right]}_{\rho }\;|\;{\left[x\right]}_{\rho }\subseteq A\right\},\\ \bar{\rho }\left(A\right)=\cup \left\{{\left[x\right]}_{\rho }\;|\;{\left[x\right]}_{\rho }\cap A\neq \emptyset \right\} \end{array}$$

are called, respectively, the *ρ*-lower and *ρ*-upper approximations of the set *A*. And $\rho (A)=\left(\underline{\rho }\left(A\right),\bar{\rho }\left(A\right)\right)$ is called a rough set with respect to *ρ*.

**Definition 4 (see [1])**

Let *ρ* be an equivalence relation on *G* and *A* a nonempty subset of *G*. Then

(2) $$\begin{array}{c} \frac{\underline{\rho }\left(A\right)}{\rho }=\left\{{\left[x\right]}_{\rho }\;|\;{\left[x\right]}_{\rho }\subseteq A\right\},\\ \frac{\bar{\rho }\left(A\right)}{\rho }=\left\{{\left[x\right]}_{\rho }\;|\;{\left[x\right]}_{\rho }\cap A\neq \emptyset \right\} \end{array}$$

are called, respectively, the lower and upper rough quotient of *A*.

**Lemma 5 (see [1])**

Let *ρ* be an equivalence relation on *G* and, for all *A*, *B*⊆*G*, one has the following.

1. $\underline{\rho }(A)\subseteq A\subseteq \bar{\rho }(A)$.
2. $\bar{\rho }\left(A\cup B\right)=\bar{\rho }\left(A\right)\cup \bar{\rho }\left(B\right)$ *;* $\bar{\rho }(A\cap B)\subseteq \bar{\rho }(A)\cap \bar{\rho }(B)$.
3. $\underline{\rho }\left(A\cup B\right)⊇\underline{\rho }\left(A\right)\cup \underline{\rho }\left(B\right)$ *;* $\underline{\rho }(A\cap B)=\underline{\rho }(A)\cap \underline{\rho }(B)$.
4. *If A*⊆*B, then* $\bar{\rho }(A)\subseteq \bar{\rho }(B)$, $\underline{\rho }(A)\subseteq \underline{\rho }(B)$.

**Lemma 6**

Let *ρ* be an equivalence relation on *G* and ∀*A*, *B*⊆*G* one has the following.

1. $\underline{\rho }(A)/\rho \subseteq A/\rho \subseteq \bar{\rho }(A)/\rho$.
2. $\bar{\rho }(A\cup B)/\rho =\bar{\rho }(A)/\rho \cup \bar{\rho }(B)/\rho$ *;* $\bar{\rho }(A\cap B)/\rho \subseteq \bar{\rho }(A)/\rho \cap \bar{\rho }(B)/\rho$.
3. $\underline{\rho }(A\cup B)/\rho ⊇\underline{\rho }(A)/\rho \cup \underline{\rho }(B)/\rho$ *;* $\underline{\rho }(A\cap B)/\rho =\underline{\rho }(A)/\rho \cap \underline{\rho }(B)/\rho$.
4. *If A*⊆*B, then* $\bar{\rho }(A)/\rho \subseteq \bar{\rho }(B)/\rho$, $\underline{\rho }(A)/\rho \subseteq \underline{\rho }(B)/\rho$.

## 3. The Homomorphism Problem of Rough Groups and Rough Quotient Groups

**Definition 7**

Let *ρ* be a complete congruence relation on *G* and *A*⊆*G*; if $\underline{\rho }(A)$ is a subgroup (a normal subgroup) of *G*, then *A* is called the lower rough group (lower rough normal subgroup) of *G* and $\underline{\rho }(A)/\rho$ is called the lower rough quotient group (lower rough normal quotient group); if $\bar{\rho }(A)$ is a subgroup (a normal subgroup) of *G*, then *A* is called the upper rough group (upper rough normal subgroup) of *G* and $\bar{\rho }(A)/\rho$ is called the upper rough quotient group (upper rough normal quotient group); if $\underline{\rho }(A),\;\bar{\rho }(A)$ are all the subgroup (normal subgroup) of *G*, then *A* is called rough group (rough normal subgroup) of *G*.

**Theorem 8**

Let *ρ* be a complete congruence relation on *G*, *A*⊆*G*, and *N*_*ρ*_⊆*A*. If *A* is a subgroup (normal subgroup) of *G*, then *A* is rough group (rough normal subgroup) of *G* and $\underline{\rho }(A)/\rho ,\;\bar{\rho }(A)/\rho$ are, respectively, lower rough quotient group (lower rough normal quotient group) and upper rough quotient group (upper rough normal quotient group).

**Corollary 9**

Let *ρ* be a complete congruence relation on *G*, *A*⊆*G*, and *N*_*ρ*_⊆*A*. Then, $\underline{\rho }(A)(\bar{\rho }(A))$ is a subgroup (normal subgroup) of *G*⇔$\underline{\rho }(A)/\rho (\bar{\rho }(A)/\rho )$ is a subgroup (normal subgroup) of *G*/*ρ*.

**Corollary 10**

Let *ρ* be a complete congruence relation on *G*, *A*⊆*G*, and *N*_*ρ*_⊆*A*. If *A* is a subgroup (normal subgroup) of *G*, then

1. $\underline{\rho }(A)<A<\bar{\rho }\left(A\right)<G$;
2. $\left(\underline{\rho }\left(A\right)⊲A⊲\bar{\rho }\left(A\right)⊲G\right)$,
3. $\underline{\rho }(A)/\rho <A/\rho <\bar{\rho }(A)/\rho <G/\rho$;
4. $\left(\underline{\rho }(A)/\rho ⊲A/\rho ⊲\bar{\rho }(A)/\rho ⊲G/\rho \right)$.

**Corollary 11**

Let *ρ* be a complete congruence relation on *G* and let *A* be a subgroup of *G* and *N*_*ρ*_⊆*A*. Then, $\bar{\rho }(A)/A≅\left(\bar{\rho }/\underline{\rho }(A))/(A/\underline{\rho }(A)\right).\;$

**Proof**

Based on the third isomorphism theorem of group, it is easy to prove this corollary.

**Lemma 12**

Let *G* and *T* be two groups; *f* : *G* → *T* is a homomorphism. If *ρ* is a congruence relation on *G* and *N*_*ρ*_⊇Ker⁡*f*, then *f*(*ρ*) is a congruence relation on *T*. Further, if *f* is an injective, then

 (1) *f*(*ρ*) = *ρ*_*f*(*N*_*ρ*_)_;
 (2) *f*(*N*_*ρ*_) = *N*_*f*(*ρ*)_,

where *f*(*ρ*) = {(*f*(*a*), *f*(*b*)) | (*a*, *b*) ∈ *ρ*}.

**Proof**

It is easy to prove *f*(*ρ*) is a congruence relation on *T*.

1. Because *N*_*ρ*_ is a normal subgroup on *G*, then *f*(*N*_*ρ*_) is the normal subgroup on *T*. So ∀(*f*(*a*), *f*(*b*)) ∈ *f*(*ρ*)⇔(*a*, *b*) ∈ *ρ*⇔*a* ∈ *bN*_*ρ*_⇔*f*(*a*) ∈ *f*(*b*)*f*(*N*_*ρ*_)⇔(*f*(*a*), *f*(*b*)) ∈ *ρ*_*f*(*N*_*ρ*_)_, so *f*(*ρ*) = *ρ*_*f*(*N*_*ρ*_)_.
2. It follows immediately from (1).

**Theorem 13**

Let *f* : *G* → *T* be an injective homomorphism. If *ρ* is a complete congruence relation on *G* and *N*_*ρ*_⊇Ker⁡*f*, then *f*(*ρ*) is a complete congruence relation on *T*.

**Proof**

According to Lemma 12, it is easy to prove *f*(*ρ*) is a congruence relation on *T*. Now we prove it is complete; ∀*a*, *b* ∈ *G*, we have [*f*(*a*)]_*f*(*ρ*)_[*f*(*b*)]_*f*(*ρ*)_ = (*f*(*a*)*f*(*N*_*ρ*_))(*f*(*b*)*f*(*N*_*ρ*_)) = *f*(*aN*_*ρ*_ *bN*_*ρ*_) = *f*(*ab*)*f*(*N*_*ρ*_) = [*f*(*ab*)]_*f*(*ρ*)_ = [*f*(*a*)*f*(*b*)]_*f*(*ρ*)_.

Therefore, *f*(*ρ*) is complete.

**Lemma 14**

Let *f* : *G* → *T* be a surjective homomorphism. If *ρ* is a congruence relation on *T*, then *f* ^−1^(*ρ*) is a congruence relation on *G*. Further, if *f* is an injective, then

1. *f* ^−1^(*ρ*) = *ρ*_*f*^−1^(*N*_*ρ*_)_;
2. *f* ^−1^(*N*_*ρ*_) = *N*_*f*^−1^(*ρ*)_,

where *f* ^−1^(*ρ*) = {(*a*, *b*) | (*f*(*a*), *f*(*b*)) ∈ *f*(*ρ*)}.

**Proof**

It is easy to prove *f* ^−1^(*ρ*) is a congruence relation on *G*.

1. Because *N*_*ρ*_ is a normal subgroup on *T*, then *f* ^−1^(*N*_*ρ*_) is the normal subgroup on *G*. So ∀(*a*, *b*) ∈ *f* ^−1^(*ρ*)⇒(*f*(*a*), *f*(*b*)) ∈ *ρ*⇒*f*(*a*) ∈ *f*(*b*)*N*_*ρ*_, and because *f* is injective, then *a* ∈ *bf* ^−1^(*N*_*ρ*_)⇒(*a*, *b*) ∈ *ρ*_*f*^−1^(*N*_*ρ*_)_; that is, *f* ^−1^(*ρ*)⊆*ρ*_*f*^−1^(*N*_*ρ*_)_. On the contrary (*a*, *b*) ∈ *ρ*_*f*^−1^(*N*_*ρ*_)_⇒*a* ∈ *bf* ^−1^(*N*_*ρ*_)⇒*f*(*a*) ∈ *f*(*b*)*N*_*ρ*_⇒(*f*(*a*), *f*(*b*)) ∈ *ρ*⇒(*a*, *b*) ∈ *f* ^−1^(*ρ*); that is, *ρ*_*f*^−1^(*N*_*ρ*_)_⊆*f* ^−1^(*ρ*); hence, *f* ^−1^(*ρ*) = *ρ*_*f*^−1^(*N*_*ρ*_)_.
2. It follows immediately from (1).

**Theorem 15**

Let *f* : *G* → *T* be an injective homomorphism. If *ρ* is a complete congruence relation on *T*, then *f* ^−1^(*ρ*) is a complete congruence relation on *G*.

**Proof**

According to Lemma 14, it is easy to prove *f* ^−1^(*ρ*) is a congruence relation on *G*. Now we prove it is complete; ∀*a*, *b* ∈ *G*, we have [*a*]_*f*^−1^(*ρ*)_[*b*]_*f*^−1^(*ρ*)_ = (*af* ^−1^(*N*_*ρ*_))(*bf* ^−1^(*N*_*ρ*_)) = *ab* *f* ^−1^(*N*_*ρ*_) = [*ab*]_*f*^−1^(*N*_*ρ*_)_.

Therefore, *f* ^−1^(*ρ*) is complete.

**Lemma 16**

Let *f* : *G* → *T* be a surjective homomorphism. If *ρ* is a congruence relation on *G*, *A*⊆*G*, and Ker⁡*f*⊆*N*_*ρ*_⊆*A*, then

1. $f(\bar{\rho }(A))=\bar{f}\left(\rho \right)\left(f\left(A\right)\right)$;
2. if *f* is injective, then $f(\underline{\rho }(A))=\underline{f}\left(\rho \right)\left(f\left(A\right)\right)$.

**Proof**

(1) Consider $\forall b\in f\left(\bar{\rho }\left(A\right)\right)\Rightarrow \exists a\in \bar{\rho }\left(A\right)$, *f*(*a*) = *b*⇒[*a*]_*ρ*_∩*A* ≠ *∅*, *f*(*a*) = *b*⇒∃*a*′ ∈ [*a*]_*ρ*_, *a*′ ∈ *A*⇒(*a*, *a*′) ∈ *ρ*, *a*′ ∈ *A*⇒(*f*(*a*), *f*(*a*′)) ∈ *f*(*ρ*), *f*(*a*′) ∈ *f*(*A*)⇒*f*(*a*′) ∈ [*f*(*a*)]_*f*(*ρ*)_, $f\left(a^{\prime }\right)\in f\left(A\right)\Rightarrow {\left[f\left(a\right)\right]}_{f(\rho )}\cap f\left(A\right)\neq \emptyset \Rightarrow f\left(a\right)=b\in \bar{f}\left(\rho \right)\left(f\left(A\right)\right)$, that is, $f(\bar{\rho }(A))\subseteq \bar{f}\left(\rho \right)\left(f\left(A\right)\right)$; on the contrary, $\forall b\in \bar{f}(\rho )(f(A))\Rightarrow \exists a\in G$, *f*(*a*) = *b*, [*b*]_*f*(*ρ*)_∩*f*(*A*) ≠ *∅*⇒∃*b*′ ∈ *f*(*A*), *b*′ ∈ [*b*]_*f*(*ρ*)_⇒∃*a*′ ∈ *A*, *f*(*a*′) = *b*′, *f*(*a*′) ∈ [*b*]_*f*(*ρ*)_⇒(*f*(*a*′), *f*(*a*)) ∈ *f*(*ρ*), *a*′ ∈ *A*⇒(*a*′, *a*) ∈ *ρ*, *a*′ ∈ *A*⇒*a*′ ∈ [*a*]_*ρ*_, $a^{\prime }\in A\Rightarrow {\left[a\right]}_{\rho }\cap A\neq \emptyset \Rightarrow a\in \bar{\rho }(A)\Rightarrow f(a)=b\in f(\bar{\rho }(A))$, that is, $f(\bar{\rho }(A))⊇\bar{f}(\rho )(f(A))$, so $f(\bar{\rho }(A))=\bar{f}(\rho )(f(A))$.

(2) Consider $\forall b\in f\left(\underline{\rho }\left(A\right)\right)\Leftrightarrow \exists a\in \underline{\rho }\left(A\right)$, *f*(*a*) = *b*⇔[*a*]_*ρ*_ = *aN*_*ρ*_⊆*A*⇔*f*([*a*]_*ρ*_) = *f*(*a*)*f*(*N*_*ρ*_)⊆*f*(*A*)⇔*bN*_*f*(*ρ*)_ = ${\left[b\right]}_{f(\rho )}\subseteq f\left(A\right)\Leftrightarrow b\in \underline{f}\left(\rho \right)\left(f\left(A\right)\right)$, so $f\left(\underline{\rho }\left(A\right)\right)$ = $\underline{f}\left(\rho \right)\left(f\left(A\right)\right)$.

**Theorem 17**

Let *f* : *G* → *T* be a surjective homomorphism, let *ρ* be a congruence relation on *G*, and let *A* be a subgroup of *G* and Ker⁡*f*⊆*N*_*ρ*_⊆*A*. Then,

1. $\bar{\rho }(A)/\rho ≅\bar{f}(\rho )(f(A))/f(\rho )$;
2. if *f* is injective, then $\underline{\rho }(A)/\rho ≅\underline{f}(\rho )(f(A))/f(\rho )$.

**Proof**

(1) Suppose that *A* is a subgroup of *G*; then $\bar{\rho }(A)$ is a subgroup on *G* and $\bar{f}(\rho )(f(A))$ is a subgroup on *T*; according to Lemma 16 $\bar{f}(\rho )(f(A))=f(\bar{\rho }(A))$; therefore, $\bar{\rho }(A)/\rho ≅\bar{f}(\rho )(f(A))/f(\rho )$.

(2) If *f* is injective, according to Lemma 16, we have $\underline{f}(\rho )(f(A))=f(\underline{\rho }(A))$; therefore, $\underline{\rho }(A)/\rho$ *≅* $\underline{f}(\rho )(f(A))/f(\rho )$.

**Corollary 18**

Let *f* : *G* → *T* be a surjective homomorphism, let *ρ* be a complete congruence relation of *G* based on Ker⁡*f*, and let *A* be a subgroup of *G* and *A*⊇Ker⁡*f*. Then,

1. $\bar{\rho }(A)/\rho ≅f(\bar{\rho }(A))$;
2. if *f* is injective, then $\underline{\rho }(A)/\rho ≅f(\underline{\rho }(A))$.

**Lemma 19**

Let *f* : *G* → *T* be a surjective homomorphism and let *ρ* be a congruence relation on *T* and *B*⊆*T*. Then

1. $f^{-1}(\bar{\rho }(B))=\bar{f^{-1}\left(\rho \right)}\left(f^{-1}\left(B\right)\right)$;
2. if *f* is an injective, then $f^{-1}\left(\underline{\rho }\left(B\right)\right)=\underline{f^{-1}\left(\rho \right)}\left(f^{-1}\left(B\right)\right)$.

**Proof**

(1) Consider $\forall a\in f^{-1}(\bar{\rho }(B))\Leftrightarrow f(a)\in \bar{\rho }(B)\Leftrightarrow {\left[f\left(a\right)\right]}_{\rho }\cap B\neq \emptyset \Leftrightarrow \exists a^{\prime }\in G$, *f*(*a*′) ∈ *B*, *f*(*a*′) ∈ [*f*(*a*)]_*ρ*_⇔*a*′ ∈ *f* ^−1^(*B*), (*f*(*a*′), *f*(*a*)) ∈ *ρ*⇔*a*′ ∈ *f* ^−1^(*B*), $\left(a^{\prime },a\right)\in f^{-1}(\rho )\Leftrightarrow {\left[a\right]}_{f^{-1}(\rho )}\cap f^{-1}(B)\neq \emptyset \Leftrightarrow a\in \bar{f^{-1}\left(\rho \right)}\left(f^{-1}\left(B\right)\right)$, and, hence, $f^{-1}\left(\bar{\rho }\left(B\right)\right)=\bar{f^{-1}\left(\rho \right)}\left(f^{-1}\left(B\right)\right)$.

(2) Consider $\forall a\in f^{-1}(\underline{\rho }(B))\Leftrightarrow f(a)\in \underline{\rho }(B)\Leftrightarrow {\left[f\left(a\right)\right]}_{\rho }$ = $f\left({\left[a\right]}_{f^{-1}(\rho )}\right)\subseteq B\Leftrightarrow {\left[a\right]}_{f^{-1}(\rho )}\subseteq f^{-1}(B)\Leftrightarrow a\in \underline{f^{-1}(\rho )}(f^{-1}(B))$, so $f^{-1}(\underline{\rho }(B))=\underline{f^{-1}(\rho )}(f^{-1}(B))$.

**Theorem 20**

Let *f* : *G* → *T* be a surjective homomorphism and let *ρ* be a congruence relation on *T* and *B* is a subgroup of *T*. Then,

1. $\bar{f^{-1}(\rho )}(f^{-1}(B))/f^{-1}(\rho )≅\bar{\rho }(B)$;
2. if *f* is an injective, then $\underline{f^{-1}(\rho )}(f^{-1}(B))/f^{-1}(\rho )≅\underline{\rho }(B)$.

**Proof**

By Lemma 19 and the first isomorphism theorem of group, we have

 
$\bar{f^{-1}(\rho )}(f^{-1}(B))/f^{-1}(\rho )=f^{-1}(\bar{\rho }(B))/f^{-1}(\rho )≅\bar{\rho }(B)$;
 
$\underline{f^{-1}(\rho )}(f^{-1}(B))/f^{-1}(\rho )=f^{-1}(\underline{\rho }(B))/f^{-1}(\rho )≅\underline{\rho }(B)$.

## 4. Congruence Relation and the Operation of Rough Group

**Lemma 21**

Let *ρ*, *ϱ* be the congruence relations on *G*. Then *N*_*ρ*∩*ϱ*_ = *N*_*ρ*_∩*N*_*ϱ*_.

**Proof**

Consider ∀*a* ∈ *N*_*ρ*∩*ϱ*_⇔(*a*, *e*) ∈ *ρ*∩*ϱ*⇔(*a*, *e*) ∈ *ρ*, (*a*, *e*) ∈ *ϱ*⇔*a* ∈ *N*_*ρ*_, *a* ∈ *N*_*ϱ*_⇔*a* ∈ *N*_*ρ*_∩*N*_*ϱ*_, and, therefore, *N*_*ρ*∩*ϱ*_ = *N*_*ρ*_∩*N*_*ϱ*_.

**Lemma 22**

Let *ρ*, *ϱ* be two congruence relations on *G* and *a* ∈ *G*. Then *a*(*N*_*ρ*_∩*N*_*ϱ*_) = *aN*_*ρ*_∩*aN*_*ϱ*_.

**Proof**

It is easy to prove that *a*(*N*_*ρ*_∩*N*_*ϱ*_)⊆*aN*_*ρ*_∩*aN*_*ϱ*_. On the contrary, ∀*b* ∈ *aN*_*ρ*_∩*aN*_*ϱ*_⇒*b* ∈ *aN*_*ρ*_, *b* ∈ *aN*_*ϱ*_⇒(*a*, *b*) ∈ *ρ*, (*a*, *b*) ∈ *ϱ*⇒(*a*, *b*) ∈ *ρ*∩*ϱ*⇒*b* ∈ *aN*_*ρ*∩*ϱ*_ = *a*(*N*_*ρ*_∩*N*_*ϱ*_); that is, *aN*_*ρ*_∩*aN*_*ϱ*_⊆*a*(*N*_*ρ*_∩*N*_*ϱ*_); therefore, *aN*_*ρ*_∩*aN*_*ϱ*_ = *a*(*N*_*ρ*_∩*N*_*ϱ*_).

**Lemma 23**

Let *ρ*, *ϱ* be two congruence relations on *G* and *a* ∈ *G*. Then [*a*]_*ρ*∩*ϱ*_ = [*a*]_*ρ*_∩[*a*]_*ϱ*_.

**Proof**

Consider ∀*b* ∈ [*a*]_*ρ*∩*ϱ*_⇔*b* ∈ *aN*_*ρ*∩*ϱ*_ = *a*(*N*_*ρ*_∩*N*_*ϱ*_) = *aN*_*ρ*_∩*aN*_*ϱ*_⇔*b* ∈ *aN*_*ρ*_, *b* ∈ *aN*_*ϱ*_⇔*b* ∈ [*a*]_*ρ*_∩[*a*]_*ϱ*_; hence [*a*]_*ρ*∩*ϱ*_ = [*a*]_*ρ*_∩[*a*]_*ϱ*_.

**Theorem 24**

Let *ρ*, *ϱ* be two complete congruence relations on *G*. Then *ρ*∩*ϱ* is a complete congruence relation on *G*.

**Proof**

Because *N*_*ρ*_, *N*_*ϱ*_ are both the normal subgroups of *G*, then *N*_*ρ*∩*ϱ*_ = *N*_*ρ*_∩*N*_*ϱ*_ is a normal subgroup of *G*, so *N*_*ρ*∩*ϱ*_ is a complete congruence relation.

**Theorem 25**

Let *ρ*, *ϱ* be two congruence relations on *G* and *A*⊆*G*. Then

1. $\bar{\rho \cap \varrho }(A)\subseteq \bar{\rho }(A)\cap \bar{\varrho }(A)$;
2. $\underline{\rho \cap \varrho }(A)⊇\underline{\rho }(A)\cup \underline{\varrho }(A)⊇\underline{\rho }(A)\cap \underline{\varrho }(A)$.

**Proof**

(1) Consider $\forall a\in \bar{\rho \cap \varrho }(A)\Rightarrow {\left[a\right]}_{\rho \cap \varrho }\cap A\neq \emptyset$. Because [*a*]_*ρ*∩*ϱ*_ = [*a*]_*ρ*_∩[*a*]_*ϱ*_, then [*a*]_*ρ*∩*ϱ*_⊆[*a*]_*ρ*_([*a*]_*ϱ*_)⇒[*a*]_*ρ*_∩*A* ≠ *∅*, ${\left[a\right]}_{\varrho }\cap A\neq \emptyset \Rightarrow a\in \bar{\rho }(A)$, $a\in \bar{\varrho }(A)\Rightarrow a\in \bar{\rho }(A)\cap \bar{\varrho }(A)$; therefore, $\bar{\rho \cap \varrho }\left(A\right)\subseteq \bar{\rho }\left(A\right)\cap \bar{\varrho }\left(A\right)$.

(2) Consider $\forall a\in \underline{\rho }(A)\cup \underline{\varrho }(A)\Rightarrow a\in \underline{\rho }(A)$ or $a\in \underline{\varrho }(A)\Rightarrow {\left[a\right]}_{\rho }\subseteq A$ or ${\left[a\right]}_{\varrho }\subseteq A\Rightarrow {\left[a\right]}_{\rho \cap \varrho }={\left[a\right]}_{\rho }\cap {\left[a\right]}_{\varrho }\subseteq A\Rightarrow a\in \underline{\rho \cap \varrho }(A)$, and, therefore $\underline{\rho \cap \varrho }(A)⊇\underline{\rho }(A)\cup \underline{\varrho }(A)⊇\underline{\rho }(A)\cap \underline{\varrho }(A)$.

**Lemma 26 (see [25])**

Let *ρ*, *ϱ* be two congruence relations on *G*. Then *ρ*∘*ϱ* is a congruence relation on *G*⇔*ρ*∘*ϱ* = *ϱ*∘*ρ*.

**Theorem 27**

Let *ρ*, *ϱ* be two complete congruence relations on *G* and *ρ*∘*ϱ* = *ϱ*∘*ρ*. Then *ρ*∘*ϱ* is a complete congruence relation on *G*.

**Proof**

By Lemma 26, *ρ*∘*ϱ* is the congruence relation and [*a*]_*ρ*∘*ϱ*_[*b*]_*ρ*∘*ϱ*_ = (*aN*_*ρ*∘*ϱ*_)(*bN*_*ρ*∘*ϱ*_) = *ab* *N*_*ρ*∘*ϱ*_ = [*ab*]_*ρ*∘*ϱ*_; hence, *ρ*∘*ϱ* is the complete congruence relation.

**Lemma 28**

Let *ρ*, *ϱ* be two congruence relations on *G*. Then *N*_*ρ*∘*ϱ*_ = *N*_*ρ*_ *N*_*ϱ*_.

**Proof**

Consider ∀*a* ∈ *N*_*ρ*∩*ϱ*_⇒(*a*, *e*) ∈ *ρ*∩*ϱ*⇒∃*b* ∈ *G*, (*a*, *b*) ∈ *ρ*, (*b*, *e*) ∈ *ϱ*⇒*a* ∈ *bN*_*ρ*_, *b* ∈ *N*_*ϱ*_⇒*ab* ∈ *bN*_*ρ*_ *N*_*ϱ*_⇒*a* ∈ *N*_*ρ*_ *N*_*ϱ*_; that is, *N*_*ρ*∘*ϱ*_⊆*N*_*ρ*_ *N*_*ϱ*_. On the contrary, ∀*a* ∈ *N*_*ρ*_ *N*_*ϱ*_⇒∃*b* ∈ *N*_*ρ*_, *c* ∈ *N*_*ϱ*_, *a* = *bc*⇒(*b*, *e*) ∈ *ρ*, (*c*, *e*) ∈ *ϱ*⇒(*bc*, *c*) ∈ *ρ*, (*c*, *e*) ∈ *ϱ*⇒(*bc*, *e*) ∈ *ρ*∘*ϱ*⇒*bc* = *a* ∈ *N*_*ρ*∘*ϱ*_; that is, *N*_*ρ*∘*ϱ*_⊇*N*_*ρ*_ *N*_*ϱ*_; hence, *N*_*ρ*∘*ϱ*_ = *N*_*ρ*_ *N*_*ϱ*_.

**Lemma 29**

Let *ρ*, *ϱ* be two congruence relations on *G* and *a*, *b* ∈ *G*. Then [*ab*]_*ρ*∘*ϱ*_ = [*a*]_*ρ*_[*b*]_*ϱ*_.

**Proof**

Consider [*ab*]_*ρ*∘*ϱ*_ = *ab* *N*_*ρ*∘*ϱ*_ = *ab* *N*_*ρ*_ *N*_*ϱ*_ = (*aN*_*ρ*_)(*bN*_*ϱ*_) = [*a*]_*ρ*_[*b*]_*ϱ*_.

**Theorem 30**

Let *ρ*, *ϱ* be two congruence relations on *G* and *ρ*∘*ϱ* = *ϱ*∘*ρ*. If *A* is a subgroup of *G* and *N*_*ρ*_, *N*_*ϱ*_⊆*A*. Then

1. $\bar{\rho \circ \varrho }(A)⊇\bar{\rho }(A)\bar{\varrho }(A)$;
2. $\underline{\rho \circ \varrho }(A)=\underline{\rho }(A)\underline{\varrho }(A)$.

**Proof**

(1) Consider $\forall a\in \bar{\rho }(A)\bar{\varrho }(A)\Rightarrow \exists b\in \bar{\rho }(A)$, $c\in \bar{\varrho }(A)$, *a* = *bc*⇒[*b*]_*ρ*_∩*A* ≠ *∅*, [*c*]_*ϱ*_∩*A* ≠ *∅*, *a* = *bc*⇒∃*b*′ ∈ [*b*]_*ρ*_, *b*′ ∈ *A*, *c*′ ∈ [*c*]_*ϱ*_, *c*′ ∈ *A*⇒*b*′*c*′ ∈ [*b*]_*ρ*_[*c*]_*ϱ*_ = [*bc*]_*ρ*∘*ϱ*_ = [*a*]_*ρ*∘*ϱ*_, $b^{\prime }c^{\prime }\in AA=A\Rightarrow {\left[a\right]}_{\rho \circ \varrho }\cap A\neq \emptyset \Rightarrow a\in \bar{\rho \circ \varrho }(A)$, so $\bar{\rho \circ \varrho }(A)⊇\bar{\rho }(A)\bar{\varrho }(A)$.

(2) Consider $\forall a\in \underline{\rho \circ \varrho }(A)\Rightarrow {\left[a\right]}_{\rho \circ \varrho }={\left[a\right]}_{\rho }{\left[e\right]}_{\varrho }\subseteq A$, because [*a*]_*ρ*_ = [*a*]_*ρ*_[*e*]⊆[*a*]_*ρ*_[*e*]_*ϱ*_⊆*A*, and ${\;\;N}_{\varrho }={\left[e\right]}_{a}\subseteq A\Rightarrow a\in \underline{\rho }\left(A\right)$, $e\in \underline{\varrho }\left(A\right)\Rightarrow a=ae\in \underline{\rho }\left(A\right)\underline{\varrho }\left(A\right)$; that is, $\underline{\rho \circ \varrho }(A)\subseteq \underline{\rho }(A)\underline{\varrho }(A)$. On the contrary, $\forall a\in \underline{\rho }(A)\underline{\varrho }(A)\Rightarrow \exists b\in \underline{\rho }(A)$, $c\in \underline{\varrho }(A)$, *a* = *bc*⇒[*b*]_*ρ*_⊆*A*, [*c*]_*ϱ*_⊆*A*, *a* = *bc*⇒[*a*]_*ρ*_∘*ϱ* = [*bc*]_*ρ*_∘*ϱ* = [*b*]_*ρ*_[*c*]_*ϱ*_⊆*AA* = $A\Rightarrow a\in \underline{\rho \circ \varrho }\left(A\right)$; that is, $\underline{\rho }(A)\underline{\varrho }(A)\subseteq \underline{\rho \circ \varrho }(A)$, so $\underline{\rho \circ \varrho }(A)=\underline{\rho }(A)\underline{\varrho }(A)$.

**Theorem 31**

Let *ρ* be complete congruence relation on *G* and *A*, *B*⊆*G*. Then

1. $\bar{\rho }(AB)=\bar{\rho }(A)\bar{\rho }(B)$;
2. $\underline{\rho }(AB)=\underline{\rho }(A)\underline{\rho }(B)$.

**Proof**

(1) Consider $\forall a\in \bar{\rho }(AB)\Rightarrow {\left[a\right]}_{\rho }\cap AB\neq \emptyset \Rightarrow \exists b\in {\left[a\right]}_{\rho }$, *b* ∈ *AB*⇒*a* ∈ *bN*_*ρ*_, ∃*c* ∈ *A*, *d* ∈ *B*, *b* = *cd*⇒*a* ∈ *cd* *N*_*ρ*_ = (*cN*_*ρ*_)(*dN*_*ρ*_) and [*c*]_*ρ*_∩*A* ≠ *∅*, [*d*]_*ρ*_∩*B* ≠ *∅*⇒∃*c*′ ∈ *N*_*ρ*_, *d*′ ∈ *dN*_*ρ*_, *a* = *c*′*d*′, because [*c*′]_*ρ*_ = [*c*]_*ρ*_, [*d*′]_*ρ*_ = [*d*]_*ρ*_⇒[*c*′]_*ρ*_∩*A* ≠ *∅*, [*d*′]_*ρ*_∩*B* ≠ *∅*, *a* = *c*′*d*′⇒*a* = $b^{\prime }c^{\prime }\in \bar{\rho }\left(A\right)\bar{\rho }\left(B\right)$; that is, $\bar{\rho }(AB)\subseteq \bar{\rho }(A)\bar{\rho }(B)$. On the contrary $\forall a\in \bar{\rho }(A)\bar{\rho }(B)\Rightarrow \exists b\in \bar{\rho }(A)$, $c\in \bar{\rho }(B)$, *a* = *bc*⇒[*b*]_*ρ*_∩*A* ≠ *∅*, [*c*]_*ρ*_∩*B* ≠ *∅*⇒∃*b*′ ∈ [*b*]_*ρ*_, *b*′ ∈ *A*, *c*′ ∈ [*c*]_*ρ*_, *c*′ ∈ *B*⇒*b*′*c*′ ∈ [*b*]_*ρ*_[*c*]_*ρ*_ = [*bc*]_*ρ*_ = [*a*]_*ρ*_, $b^{\prime }c^{\prime }\in AB\Rightarrow {\left[a\right]}_{\rho }\cap AB\neq \emptyset \Rightarrow a\in \bar{\rho }\left(AB\right)$; that is, $\bar{\rho }(A)\bar{\rho }(B)\subseteq \bar{\rho }(AB)$. Therefore $\bar{\rho }(AB)=\bar{\rho }(A)\bar{\rho }(B)$.

(2) $\;\forall a\in \underline{\rho }(AB)\Rightarrow {\left[a\right]}_{\rho }\subseteq AB\Rightarrow \exists b\in A,c\in B,a=bc\Rightarrow {\left[b\right]}_{\rho }{\left[c\right]}_{\rho }={\left[bc\right]}_{\rho }={\left[a\right]}_{\rho }\subseteq AB$, if ∃*b*′ ∈ [*b*]_*ρ*_, *b*′∋*A*, *c*′ ∈ [*c*]_*ρ*_, *c*′∋*B*, then *b*′*c*′ ∈ [*b*]_*ρ*_[*c*]_*ρ*_, *b*′*c*′∋*AB*, Contradiction, that is, [*b*]_*ρ*_⊆*A*, [*c*]_*ρ*_⊆*B*, so, $a=bc\in \underline{\rho }\left(A\right)\underline{\rho }\left(B\right)$, that is, $\underline{\rho }(AB)\subseteq \underline{\rho }(A)\underline{\rho }(B)$. On the contrary, $\forall a\in \underline{\rho }(A)\underline{\rho }(B)\Rightarrow \exists b\in \underline{\rho }(A)$, $c\in \underline{\rho }(B)$, *a* = *bc*⇒[*b*]_*ρ*_⊆*A*, [*c*]_*ρ*_⊆*B*, $a=bc\Rightarrow {\left[a\right]}_{\rho }={\left[bc\right]}_{\rho }={\left[b\right]}_{\rho }{\left[c\right]}_{\rho }\subseteq AB\Rightarrow a\in \underline{\rho }(AB)$, that is, $\underline{\rho }\left(A\right)\underline{\rho }\left(B\right)\subseteq \underline{\rho }\left(AB\right)$, so, $\underline{\rho }(AB)=\underline{\rho }(A)\underline{\rho }(B)$.

**Corollary 32**

Let *ρ* be a complete congruence relation on *G* and *A*⊆*G*, *n* ∈ *N*. Then

1. $\bar{\rho }(A^{n})={\left(\bar{\rho }\left(A\right)\right)}^{n}$;
2. $\underline{\rho }(A^{n})={\left(\underline{\rho }\left(A\right)\right)}^{n}$.

**Corollary 33**

Let *ρ* be a complete congruence relation on *G* and let *A* be a subgroup of *G* and *n* ∈ *N*. Then

1. ${\bar{\rho }}^{n}(A)={\left(\bar{\rho }\left(A\right)\right)}^{n}=\bar{\rho }(A^{n})=\bar{\rho }(A)$;
2. ${\underline{\rho }}^{n}(A)={\left(\underline{\rho }\left(A\right)\right)}^{n}=\underline{\rho }(A^{n})=\underline{\rho }(A)$.

**Proof**

Because *A* is a subgroup, then $\bar{\rho }(A),\underline{\rho }(A)$ are both the subgroups of *G*, so ${\left(\bar{\rho }\left(A\right)\right)}^{n}=\bar{\rho }(A)$, ${\left(\underline{\rho }\left(A\right)\right)}^{n}=\underline{\rho }(A)$, *A* ^*n*^ = *A*, and *ρ* is an equivalence relation; then *ρ* ^*n*^ = *ρ*; hence, we can get (1) and (2).

**Corollary 34**

Let *ρ* be a complete congruence relation on *G* and let *A*, *B* be two subgroups of *G*. Then

1. $\bar{\rho }(A\cup B)=\bar{\rho }(A)\cup \bar{\rho }(B)\subseteq \bar{\rho }(AB)=\bar{\rho }(A)\bar{\rho }(B)$;
2. $\underline{\rho }(A\cup B)\subseteq \underline{\rho }(AB)=\underline{\rho }(A)\underline{\rho }(B)$.

**Proof**

(1) Because *A* = *A*(*e*)⊆*AB*, and *B*⊆*AB*, so *A* ∪ *B*⊆*AB*; hence, $\bar{\rho }(A\cup B)\subseteq \bar{\rho }(AB)$ and $\bar{\rho }\left(A\cup B\right)=\bar{\rho }\left(A\right)\cup \bar{\rho }\left(B\right)$; therefore, (1) holds. (2) Because *A* ∪ *B*⊆*AB*, so $\underline{\rho }(A\cup B)\subseteq \underline{\rho }(AB)=\underline{\rho }(A)\underline{\rho }(B)$.
